# Comparative Dosimetric and Radiobiological Evaluation of Dental Structures and Normal Tissue Complication Probability

**DOI:** 10.7759/cureus.109654

**Published:** 2026-05-25

**Authors:** Sunil Kumar Gulia, Jasmine Kalsi, Satish D Mehta, Rahul Tiwari, Raj K Tiwari, Unni J Appilly

**Affiliations:** 1 Department of Oral and Maxillofacial Surgery, Shree Guru Gobind Singh Tricentenary University, Gurugram, IND; 2 Department of General Surgery, Kalpana Chawla Government Medical College, Karnal, IND; 3 Department of Orthopaedics, Bharati Vidyapeeth (Deemed to be University) Medical College, Sangli, IND; 4 Department of Oral and Maxillofacial Surgery, Narsinhbhai Patel Dental College and Hospital, Visnagar, IND; 5 Department of Oral and Maxillofacial Surgery, Hitkaarni Dental College and Hospital, Jabalpur, IND; 6 Department of Prosthodontics, University of Otago, Dunedin, NZL

**Keywords:** dental structures, dose-volume histogram, head and neck cancer, normal tissue complication probability, radiotherapy

## Abstract

Introduction: Head and neck cancers require highly conformal radiotherapy techniques to achieve optimal tumor control while minimizing damage to the surrounding critical structures. The present study aimed to compare advanced radiotherapy planning techniques with an emphasis on dental structures and radiobiological outcomes.

Materials and methods: This retrospective observational dosimetric study was conducted on radiotherapy planning records of 25 patients treated over a 10-year period with head and neck malignancies. For each patient, two radiotherapy plans were generated on the same computed tomography dataset using identical optimization parameters. Target volumes and organs at risk (OAR), including the dental structures, parotid glands, mandible, and spinal cord, were contoured. Dose-volume histogram parameters, such as the mean dose, maximum dose, and volume-based indices, were analyzed. Radiobiological evaluation was performed using normal tissue complication probability (NTCP) modeling for xerostomia and mandibular osteoradionecrosis. Statistical analysis was performed using paired tests with a significance level of less than 0.05.

Results: Both techniques achieved comparable target volume coverage; however, significant improvements in plan quality were observed with the arc-based delivery. The conformity index was higher (0.87 ± 0.04 versus 0.82 ± 0.05; p < 0.001), and the homogeneity index was improved (0.078 ± 0.015 vs. 0.091 ± 0.018; p < 0.001). Mean dose to the parotid glands was significantly reduced (24.6 ± 3.8 gray versus 29.8 ± 4.2 gray; p < 0.001). Dental structures demonstrated lower mean dose (30.4 ± 8.1 gray versus 36.1 ± 8.9 gray; p = 0.002) and reduced volume receiving 30 gray (40.8 ± 13.1% versus 50.4 ± 14.2%; p = 0.001). The mandibular dose parameters were also significantly reduced (p = 0.001). Radiobiological analysis showed a significant reduction in predicted xerostomia (27.3 ± 9.8% versus 38.4 ± 11.6%; p < 0.001) and mandibular osteoradionecrosis (5.2 ± 3.9% vs. 8.7 ± 5.4%; p < 0.001).

Conclusion: Advanced radiotherapy planning techniques provide superior dose conformity, improved sparing of dental structures and OAR, and a significant reduction in predicted complication probabilities. These findings support their clinical use for optimizing treatment outcomes and improving the long-term quality of life in patients with head and neck cancer.

## Introduction

Head and neck cancers represent a major global health burden, with disproportionately high incidence and morbidity in countries such as India, where oral malignancies are among the most prevalent cancers [1,2]. Radiotherapy plays a central role in the management of these tumors either as a definitive modality or in combination with surgery and chemotherapy. Over the past two decades, technological advancements, such as intensity-modulated radiotherapy (IMRT) and volumetric modulated arc therapy (VMAT), have revolutionized treatment delivery by enabling highly conformal dose distributions while minimizing radiation exposure to surrounding normal tissues [3,4].

IMRT has long been established as the standard of care because of its ability to modulate radiation intensity across multiple fixed beams, thereby improving target coverage and reducing toxicity to organs at risk (OARs) [5]. However, VMAT, a more recent innovation, delivers radiation through continuous gantry rotation with the dynamic modulation of multileaf collimators, dose rate, and gantry speed, offering potential advantages in dose conformity, treatment efficiency, and OAR sparing [6]. While numerous comparative studies have focused on conventional OARs, such as prostate cancer, parotid glands, and spinal cord, relatively limited attention has been paid to dental structures, which are highly susceptible to radiation-induced damage [4,7,8].

Radiation exposure to teeth and jawbones can result in significant complications, including radiation caries, enamel demineralization, pulp necrosis, and osteoradionecrosis, all of which adversely affect long-term quality of life [9]. With increasing emphasis on survivorship and functional outcomes, there is a growing need to evaluate not only dosimetric differences but also the radiobiological consequences of treatment techniques. In this context, normal tissue complication probability (NTCP) modeling provides a clinically meaningful framework for estimating the risk of complications such as xerostomia and mandibular osteoradionecrosis based on dose-volume parameters [10].

The present study aimed to perform a comprehensive comparative evaluation of IMRT and VMAT in patients with head and neck cancer by integrating dosimetric and radiobiological analyses. The objectives were to (1) compare dose-volume parameters for dental structures and conventional OARs, (2) evaluate target volume coverage and plan quality indices, (3) assess treatment delivery efficiency, and (4) estimate and compare NTCP for xerostomia and mandibular osteoradionecrosis between the two techniques. The primary endpoint was the paired difference in predicted xerostomia NTCP between IMRT and VMAT, while secondary endpoints included mandibular osteoradionecrosis NTCP, dose-volume histogram parameters of dental structures and OAR, target coverage indices, and treatment planning quality metrics.

## Materials and methods

Study design

This retrospective observational dosimetric study was conducted in June 2023 in the Department of Oral and Maxillofacial Surgery at Narsinhbhai Patel Dental College and Hospital, Visnagar, India. The study utilized anonymized radiotherapy planning records of 25 patients treated over a 10-year period, from January 2012 to December 2021. As the investigation was based exclusively on previously recorded and de-identified data without any direct patient interaction or intervention, the requirement for ethical approval and informed consent was waived in accordance with the institutional guidelines for retrospective studies by the Institutional Ethical Committee of Narsinhbhai Patel Dental College and Hospital (approval number: NPDCH/IEC/2023/192). This study was conducted in compliance with the ethical principles outlined in the Declaration of Helsinki.

Inclusion and exclusion criteria

Patient datasets were retrieved from the institutional archives and screened for eligibility. Patients with histologically confirmed head and neck malignancies involving regions adjacent to dental structures, including the oral cavity and oropharynx, were included in the study. Only cases with complete, high-quality planning computed tomography datasets, intact dental and alveolar structures within the irradiated field, and comprehensive treatment planning data were included. Patients with prior radiotherapy to the head and neck region, incomplete datasets, or significant imaging artifacts such as metallic dental restorations affecting dose calculation accuracy were excluded. A total of 25 patients fulfilling these criteria were included in the final analysis. A paired study design was employed, wherein two separate treatment plans were generated for each patient on the same dataset, thereby eliminating inter-patient anatomical variability and enabling robust intra-patient comparisons.

Study participants

All patients had previously undergone computed tomography simulation in the supine position using a thermoplastic immobilization system (Klarity Medical Products, Newark, OH, USA) to ensure reproducibility and setup accuracy. Planning CT scans were acquired using a dedicated radiotherapy simulator (GE LightSpeed RT16, GE Healthcare, Chicago, IL, USA) with a slice thickness of 2.5-3 mm, extending from the vertex to the carina. The imaging datasets were subsequently transferred to a commercial treatment planning system (Eclipse™ Version 15.6, Varian Medical Systems, Palo Alto, CA, USA) for contouring and plan generation. Dose calculations were performed using an Anisotropic Analytical Algorithm integrated into the planning system. All treatment plans were generated for delivery on a linear accelerator (Clinac iX, Varian Medical Systems) equipped with a Millennium 120 multileaf collimator to ensure clinical deliverability.

Target volumes, including gross tumor volume, clinical target volume, and planning target volume, were delineated in accordance with the established international recommendations [11]. OARs, including the bilateral parotid glands, spinal cord, oral cavity, and mandible, were contoured using standardized institutional protocols. Dental structures were delineated as a composite volume encompassing the maxillary and mandibular teeth along with the surrounding alveolar bone, using bone window settings to improve contouring precision. All contours were based on clinically approved datasets without re-contouring, ensuring consistency and minimizing observer bias.

For each patient, two radiotherapy plans representing advanced delivery techniques were generated on the same CT dataset using identical optimization objectives, dose constraints, and priority settings to ensure an unbiased comparison. Treatment plans were normalized such that at least 95% of the planning target volume received 95% of the prescribed dose, which ranged from 60 to 70 Gy delivered in 30-35 fractions depending on the clinical indication.

Outcome analysis

Dosimetric evaluations were performed using dose-volume histograms extracted from the treatment planning system. Target coverage parameters, including near-minimum dose (D98%), near-prescription dose (D95%), and near-maximum dose (D2%), were recorded. The plan quality was assessed using conformity and homogeneity indices. For OAR, the mean dose (Dmean), maximum dose (Dmax), and clinically relevant volume-based parameters such as V26Gy, V30Gy, V40Gy, V50Gy, and V60Gy were analyzed. Particular emphasis was placed on dental structures, for which the mean dose, maximum dose, and volume-based thresholds were evaluated due to their established association with radiation-induced dental complications.

Radiobiological evaluation was performed using NTCP modeling based on the Lyman-Kutcher-Burman model [12-15]. The probability of xerostomia was estimated using dose-volume parameters of the combined parotid glands, while the risk of mandibular osteoradionecrosis was calculated using mandibular dose distributions. Equivalent uniform dose values were derived from dose-volume histogram data, and NTCP values were computed using the established model parameters reported in the literature. No clinical outcome data were used because this was a purely dosimetric and modeling-based study.

Sample size estimation

The sample size for this paired dosimetric comparison was determined based on the primary endpoint of the predicted normal tissue complication probability difference between the two radiotherapy planning techniques, specifically for xerostomia. A priori power analysis was performed using the G*Power software (version 3.1, Heinrich Heine University, Düsseldorf, Germany). A medium effect size (Cohen’s d = 0.5) was assumed for the paired difference in NTCP values based on existing literature [16]. With a two-sided alpha level of 0.05 and a statistical power of 80%, the minimum required sample size was calculated as 23 patients. To account for potential technical failures during plan generation or incomplete dose-volume histogram data extraction, the target sample size was increased to 25 patients, thereby ensuring adequate statistical robustness and reliability of the analysis.

Statistical analysis

Statistical analysis was performed using IBM SPSS Statistics (version 26.0, IBM Corp., Armonk, NY, USA) and R statistical software (version 4.2, R Foundation for Statistical Computing, Vienna, Austria). Continuous variables are expressed as mean ± standard deviation. The normality of paired differences was assessed using the Shapiro-Wilk test. As the study design was paired, comparisons between the two techniques were performed using paired t-tests for normally distributed variables, with Bonferroni correction applied for multiple comparisons, where indicated. An exploratory subgroup analysis examined the absolute NTCP reduction in patients with baseline IMRT osteoradionecrosis risk exceeding 5%, reported as median with interquartile range. No imputation for missing data was required, as all 25 patients had complete dose-volume histogram datasets. A two-sided p-value of less than 0.05 was considered statistically significant.

## Results

A total of 25 patients were included in the paired dosimetric analysis (Table 1). The cohort comprised 18 (72%) males and seven (28%) females, with a median age of 58 years (IQR: 49-66 years). The most common primary tumor site was the oropharynx, followed by the oral cavity. Most patients presented with advanced disease. The median prescribed dose to the high-risk planning target volume was 70 Gy. Normality testing confirmed that all paired differences were normally distributed (p > 0.05), supporting the use of parametric analysis.

**Table 1:** Patient demographics, tumor characteristics, and planning computed tomography parameters (n = 25). Continuous variables are presented as median with interquartile range, and categorical variables as count and percentage. Descriptive statistics were used; no inferential statistical test was applied for this table.

Characteristic	Categories	Data values
Sex, n (%)	Male	18 (72%)
	Female	7 (28%)
Age at planning, median (interquartile range)	Years	58 (49–66)
Primary tumour site, n (%)	Oropharynx	12 (48%)
	Oral cavity	6 (24%)
	Larynx/Hypopharynx	5 (20%)
	Nasopharynx	2 (8%)
Tumor stage, n (%)	T3	8 (32%)
	T4	17 (68%)
Nodal stage, n (%)	N2	5 (20%)
	N3	20 (80%)
Planning parameters, median (interquartile range)	Prescribed dose to high-risk planning target volume, gray	70 (66-78)
	Number of fractions	35 (33-38)
	Computed tomography slice thickness, millimeters	2.5 (1.25–2.5)

VMAT demonstrated significantly superior planning target volume coverage compared to IMRT. The conformity index was higher (0.87 ± 0.04 vs 0.82 ± 0.05; mean difference +0.05, p < 0.001), while the homogeneity index was lower (0.08 ± 0.02 vs 0.09 ± 0.01; mean difference −0.01, p < 0.001). Additionally, VMAT achieved a higher volume receiving 95% dose (99.10 ± 0.70% vs 98.70 ± 0.80%; p = 0.008) and a lower volume receiving 107% dose (0.90 ± 0.50% vs 1.40 ± 0.60%; p < 0.001) (Table 2).

**Table 2:** Comparison of planning target volume coverage indices between techniques. Comparisons were performed using a paired t-test after confirmation of normal distribution using the Shapiro–Wilk test. Higher conformity index and lower homogeneity index indicate superior plan quality. Mean differences are expressed as volumetric modulated arc therapy minus intensity-modulated radiotherapy, df: degree of freedom, *p < 0.05 denotes statistical significance.

Parameter	Intensity-modulated radiotherapy (mean ± standard deviation)	Volumetric modulated arc therapy (mean ± standard deviation)	Mean difference (95% confidence interval)	t (df = 24)	p-value
Conformity index (CI)	0.82 ± 0.05	0.87 ± 0.04	+0.05 (+0.03 to +0.07)	5.31	< 0.001*
Homogeneity index (HI)	0.09 ± 0.01	0.08 ± 0.02	−0.01 (−0.02 to −0.01)	4.57	< 0.001*
Volume receiving 95% dose (%)	98.70 ± 0.80	99.10 ± 0.70	+0.40 (+0.10 to +0.70)	2.89	0.008*
Volume receiving 107% dose (%)	1.40 ± 0.60	0.90 ± 0.50	−0.50 (−0.70 to −0.30)	5.00	< 0.001*

Significant improvements in OAR sparing were observed using VMAT (Table 3). The mean dose to the combined parotid glands was significantly reduced (24.6 ± 3.8 Gy vs. 29.8 ± 4.2 Gy; p < 0.001), along with reductions in V26Gy and V30Gy (both p < 0.001). Mandibular dose parameters were also significantly lower with VMAT, including Dmean (33.2 ± 6.9 Gy vs. 38.4 ± 7.6 Gy; p = 0.001) and V50Gy (33.7 ± 11.6% vs. 41.3 ± 12.4%; p = 0.001). For dental structures, VMAT demonstrated significantly reduced Dmean (30.4 ± 8.1 Gy vs. 36.1 ± 8.9 Gy; p = 0.002) and V30Gy (40.8 ± 13.1% vs. 50.4 ± 14.2%; p = 0.001). No statistically significant difference was observed in the spinal cord Dmax (p = 0.096).

**Table 3:** Comparison of organ-at-risk dosimetric parameters between techniques. Comparisons were performed using a paired t-test after confirmation of normal distribution using the Shapiro–Wilk test. Mean dose refers to the average radiation dose received by the structure. Volume receiving x gray represents the percentage of volume receiving at least that dose, df: degree of freedom, *p < 0.05 denotes statistical significance.

Structure	Parameter	Intensity-modulated radiotherapy (mean ± standard deviation)	Volumetric modulated arc therapy (mean ± standard deviation)	Mean difference (95% confidence interval)	t (df = 24)	p-value
Parotid glands (combined)	Mean dose (gray)	29.8 ± 4.2	24.6 ± 3.8	−5.2 (−6.8 to −3.6)	6.74	< 0.001*
	Volume receiving 26 gray (%)	54.3 ± 9.6	43.1 ± 8.7	−11.2 (−14.9 to −7.5)	6.19	< 0.001*
	Volume receiving 30 gray (%)	47.2 ± 10.1	36.4 ± 9.2	−10.8 (−14.7 to −6.9)	5.65	< 0.001*
Mandible	Maximum dose (gray)	70.1 ± 3.4	67.3 ± 3.1	−2.8 (−4.3 to −1.3)	3.84	0.001*
	Mean dose (gray)	38.4 ± 7.6	33.2 ± 6.9	−5.2 (−7.9 to −2.5)	3.97	0.001*
	Dose to 1 cubic centimeter (gray)	65.7 ± 5.1	61.8 ± 4.7	−3.9 (−5.8 to −2.0)	4.24	<0.001*
	Volume receiving 50 gray (%)	41.3 ± 12.4	33.7 ± 11.6	−7.6 (−11.9 to −3.3)	3.62	0.001*
	Volume receiving 60 gray (%)	29.8 ± 10.8	22.6 ± 9.7	−7.2 (−11.1 to −3.3)	3.78	0.001*
Dental structures	Mean dose (gray)	36.1 ± 8.9	30.4 ± 8.1	−5.7 (−9.0 to −2.4)	3.57	0.002*
	Maximum dose (gray)	68.3 ± 4.2	65.1 ± 4.0	−3.2 (−4.9 to −1.5)	3.88	0.001*
	Volume receiving 30 gray (%)	50.4 ± 14.2	40.8 ± 13.1	−9.6 (−15.0 to −4.2)	3.67	0.001*
	Volume receiving 40 gray (%)	38.7 ± 13.5	29.5 ± 12.3	−9.2 (−14.3 to −4.1)	3.72	0.001*
Spinal cord	Maximum dose (gray)	43.2 ± 3.8	42.1 ± 3.5	−1.1 (−2.4 to +0.2)	1.73	0.096

Radiobiological analysis revealed significant reductions in the predicted NTCP with VMAT (Table 4). The estimated risk of xerostomia was significantly lower with VMAT than with IMRT (27.3 ± 9.8% vs. 38.4 ± 11.6%; p < 0.001), corresponding to an absolute reduction of 11.1%. Similarly, the predicted risk of mandibular osteoradionecrosis was reduced from 8.7 ± 5.4% to 5.2 ± 3.9% (p < 0.001), representing a mean reduction of 3.5%.

**Table 4:** Comparison of predicted normal tissue complication probability between techniques. Comparisons were performed using paired t-test after confirmation of normal distribution using Shapiro–Wilk test, Predicted normal tissue complication probability values were calculated using the Lyman-Kutcher-Burman model based on dose–volume histogram data, df: degree of freedom, *p < 0.05 denotes statistical significance.

Endpoint	Intensity-modulated radiotherapy (mean ± standard deviation, %)	Volumetric modulated arc therapy (mean ± standard deviation, %)	Mean difference (95% confidence interval, %)	t (df = 24)	p-value
Xerostomia (stimulated saliva <25% at 12 months)	38.4 ± 11.6	27.3 ± 9.8	−11.1 (−14.8 to −7.4)	6.12	< 0.001*
Mandibular osteoradionecrosis	8.7 ± 5.4	5.2 ± 3.9	−3.5 (−5.1 to −1.9)	4.51	< 0.001*

## Discussion

The present study provides a comprehensive comparative evaluation of advanced radiotherapy planning techniques for head and neck cancer, integrating both dosimetric and radiobiological perspectives, with a specific focus on dental structures. The findings demonstrated that while target volume coverage was comparable between the two techniques, significant improvements were observed in plan quality, OAR sparing, and predicted normal tissue complication probability.

In terms of target coverage, both techniques achieved clinically acceptable dose distributions, consistent with the established radiotherapy standards. However, the significantly improved conformity and homogeneity indices observed with VMAT indicated superior dose shaping and uniformity. These findings align with those of an earlier study that demonstrated enhanced dose conformity with arc-based delivery compared with fixed-field techniques [3]. Improved conformity is clinically relevant, as it reduces unnecessary irradiation of the surrounding healthy tissues without compromising tumor coverage. Similarly, the reduction in high-dose regions (V107%) observed in this study suggests improved control of dose hotspots, which may contribute to the reduced toxicity.

A key strength of this study lies in its detailed evaluation of dental structures, which remains an underexplored OAR in radiotherapy planning [17]. The significant reduction in mean dose and volume-based parameters for dental tissues observed with advanced radiotherapy techniques is clinically important, as it aligns with previous studies demonstrating improved dose conformity and OAR sparing with arc-based delivery approaches without compromising target coverage [18,19]. The present findings suggest that improved dose modulation with VMAT may contribute to better preservation of dental structures, which is particularly relevant for patients with long-term survival.

In addition to dental structures, significant reductions in the dose to conventional OAR, particularly to the parotid glands and mandible, were observed. The reduction in the parotid mean dose and associated volume parameters is consistent with findings from the trial by Nutting et al., which established the clinical importance of salivary gland sparing in reducing xerostomia [20]. Similarly, the improved mandibular dose parameters observed in this study are clinically significant, given the well-established association between high mandibular dose and the risk of osteoradionecrosis. A previous study by Zhang et al. [21] also demonstrated improved organ sparing using arc-based techniques, supporting the consistency of the present findings.

A notable contribution of this study is the incorporation of radiobiological modeling through NTCP analysis, which provides a clinically meaningful estimation of the complication risk beyond conventional dosimetric parameters. The significant reduction in the predicted xerostomia risk with VMAT is consistent with the observed reduction in parotid dose and supports the clinical relevance of dosimetric improvements [20]. Similarly, the reduction in predicted mandibular osteoradionecrosis risk highlights the potential of advanced planning techniques to minimize severe late complications. NTCP modeling represents an important step toward personalized and outcome-based radiotherapy planning [16].

These findings have important clinical implications. The improved sparing of dental structures and reduction in predicted complication probabilities suggest that advanced radiotherapy techniques may enhance long-term oral health outcomes and the overall quality of life in head and neck cancer survivors. Given the increasing emphasis on survival and functional outcomes, incorporating dental structures into routine treatment planning and evaluation may be beneficial. Furthermore, improved dose conformity and reduced treatment-related toxicity may facilitate better treatment compliance and reduce the need for supportive care.

Despite these strengths, certain limitations must be acknowledged. The retrospective design of the study introduces the possibility of selection and information bias, and the relatively small sample size derived from a single institution and limited geographical region may restrict the generalizability of the findings. In addition, the study population predominantly consisted of older adults, who may present with systemic comorbidities and age-related physiological variations that could potentially influence radiobiological responses and treatment-related toxicity. Although the paired intra-patient design minimized anatomical variability, the use of clinically approved contours without re-contouring or formal inter-observer variability assessment may have introduced contouring-related bias. Variations in the delineation of dental structures and adjacent organs-at-risk could affect dose-volume histogram parameters and consequently influence NTCP estimations. Furthermore, while identical normalization criteria were applied to both planning techniques to maintain consistency in comparison, different normalization approaches may alter absolute dosimetric outcomes. The study was also based primarily on dosimetric and radiobiological modeling without direct prospective clinical correlation of xerostomia severity, dental complications, or mandibular osteoradionecrosis. Although NTCP models provide clinically meaningful predictive estimates, they remain dependent on underlying model assumptions and may not fully account for individual patient variability. Therefore, the observed reductions in predicted complication probability should be interpreted cautiously and require validation through larger prospective multicenter studies with long-term clinical follow-up.

## Conclusions

Within the limitations of this retrospective dosimetric and radiobiological analysis, advanced radiotherapy planning has demonstrated superior performance in head and neck cancer treatment. While target volume coverage was comparable, significant improvements were observed in dose conformity, homogeneity, and sparing of critical structures, particularly in the dental tissues, parotid glands, and mandible. Importantly, these dosimetric advantages translate into meaningful reductions in the predicted probability of normal tissue complications for xerostomia and mandibular osteoradionecrosis. These findings highlight the clinical relevance of incorporating dental structures into treatment planning and support the preferential use of advanced delivery techniques to optimize the therapeutic ratio and improve long-term patient outcomes.
